# Caesarean deliveries in China

**DOI:** 10.1186/s12884-017-1233-8

**Published:** 2017-02-06

**Authors:** Xin Wang, Susan Hellerstein, Lei Hou, Liying Zou, Yan Ruan, Weiyuan Zhang

**Affiliations:** 1 Department of Obstetrics, Beijing Obstetrics and Gynecology Hospital, Capital Medical University, No. 251 YaoJiayuan Road, Chaoyang District, Beijing, 100026 China; 2 Department of Obstetrics and Gynecology, Brigham and Women’s Hospital, Harvard Medical School, Boston, MA 01210 USA

**Keywords:** Caesarean section, Indication, Tertiary level hospital, Secondary level hospital

## Abstract

**Background:**

The caesarean section rate has risen rapidly in China. The purpose of this retrospective study was to estimate caesarean section rates and indications by hospital facility level in Mainland China to investigate reasons contributing to the high rate.

**Methods:**

This cross-sectional hospital-based study collected data from 39 hospitals in three geographical regions in China, covering 14 different provinces, municipalities, and autonomous regions, including 20 tertiary health hospitals and 19 secondary hospitals. Data from all women who gave birth at these hospitals during 2011 were included.

**Results:**

A total of 112,138 women who gave birth after 24 weeks of gestation were surveyed. Of these pregnancies, 54.5% (61,084 cases) resulted in caesarean section, non-indicated caesarean section accounted for 38.4% of caesarean sections. Overall caesarean section rates were higher at the tertiary level hospitals (55.9%) compared to the secondary level hospitals (50.9%). The secondary level hospitals had higher rates of non-indicated caesarean section (48.9% of caesarean sections) compared to tertiary level hospitals (34.5% of caesarean sections). The rate of caesarean section on maternal request was higher in the secondary hospitals (16.6%) than in the tertiary hospitals (10%) (*P* < 0.001), as well as the caesarean section rate for CPD prior to labour. Operative vaginal deliveries were overall rare (1.2%) with 90.9% (1200/1320 cases) performed in the tertiary hospitals.

**Conclusions:**

Caesarean section on maternal request accounts for a large portion of China’s high caesarean section rate, especially in the secondary hospitals. The first step to reduced caesarean section rates is to decrease the number of non-indicated caesarean sections.

## Background

The caesarean section rate has risen rapidly worldwide in recent decades and is a global concern [1–6]. A World Health Organization (WHO) survey from 2004 to 2008 reported a 25.7% average global caesarean section rate, with 27.3% in Asia, 19.0% in Europe and 29.2% in Latin America [1, 2]. China had the highest overall caesarean section rate (46.2%) of the 24 countries in the survey [1]. Over the past decades, the caesarean section rate in China increased sharply for women in all regions, from all socio-economic groups, and in all levels of hospitals [7]. Huang reported that the caesarean section rate increased from 8.9% in 1993–1994 to 24.8% in 2001–2002 [8]. Feng reported that 64.1% of urban women and 11.3% of women in the poorest rural region gave birth by caesarean section in 2008 [9]. Between 1993 and 2008, the risk of caesarean section increased more than three times in urban areas and more than 15-fold in rural areas in China [9]. A recent study of one region found a caesarean section rate of 80% [10]. The overuse of caesarean section has become an important public health problem in China.

It has been suggested that non-indicated caesarean sections are among the main drivers of the high caesarean section rate [1, 8, 9]. A study of 56,968 caesarean sections in southern China showed that the prevalence of caesarean section during 1993–1995, 1996–2000, and 2001–2005 was 13.1, 28.3, and 50.4%, respectively and that the prevalence of caesarean section on maternal request was 0.6, 3.8, and 12.9%, respectively [7]. In the above mentioned WHO study, the overall proportion of women delivering by non-indicated caesarean section ranged from 0.01 to 2.10%, except in China, where this figure was exceptionally high at 11.6% [1].

In China, hospitals are classified into three groups: primary, secondary and tertiary level hospitals according to their service level, size, medical technology, medical equipment, management and medical quality. The tertiary level hospitals provide the highest level of medical service. Primary care facilities include hospitals and community-based health care facilities that provide preventive care, and other basic community services but no delivery care.

Little is known about the actual rate of caesarean section in China. Previous studies have been limited to several hospitals or regions. Information about mode of delivery and indications for caesarean section at secondary and tertiary facilities needs analysis to understand the high caesarean section rates in China. The goal of this study is to compare mode of delivery and indications for caesarean section in secondary and tertiary level hospitals to better understand reasons for China’s high caesarean section rate.

## Methods

### Study design and subjects

In this multi-center cross-sectional study, discharge data were collected from all live births from January 1, 2011 through December 31, 2011in 39 public hospitals from14 provinces of China. All participating facilities are members of an obstetrics cooperative center, with broader medical and academic collaboration.

The hospitals included were all part of the public system, since in 2011 more than 95% of deliveries occurred at public hospitals. The 14 provinces, municipalities, and autonomous regions within China included hospitals from Beijing, Shanghai, Jilin, Liaoning, Jiangsu, Sichuan, Shanxi, Hubei, Guangdong, Hebei, Inner Mongolia, Shandong, Shanxi, and Xinjiang.

There were twenty tertiary care hospitals and nineteen secondary care hospitals from 3 regions: East China, Central China, and West China. In China by 2011 98.7% of deliveries took place in hospitals [11] and in 2012, 94.4% of all inpatients in China were admitted to secondary and tertiary level hospitals [12]. Most primary care hospitals do not have obstetrics departments or neonatal care services and are not equipped to perform caesarean section. Therefore, primary care hospitals were not included in the present study. This study was not able to study urban versus rural patient origin.

All individual-level data obtained from medical records were coded in a de-identified format, thus patient consent was not required. The investigators had no contact with patients. The procedures of this study received approval from the Human Ethics Committees of every participating hospital.

### Data collection

All women who had live births in the calendar year 2011 at each hospital site were included in the data collection. There was uniform face-to-face training on data extraction for the physician coordinator at each site. At the end of 2011, the data were extracted from the medical records and discharge summaries by trained medical staff on a standardized coded form for computer-based statistical analysis. The data points included: demographics, maternal data (age, parity, education, medical comorbidities), obstetric factors (gestational age, presentation, gestational diabetes, preeclampsia, premature rupture of membranes, third trimester bleeding), mode of delivery, and indication for caesarean section. The physician-documented indication for caesarean section was recorded. If there was more than one indication the physician designated primary indication was used.

### Data analysis

Vaginal birth, operative vaginal birth and overall caesarean section rates were calculated for the total sample and for secondary and tertiary level facilities. Caesarean section was divided into two categories: indicated and non-indicated. The indicated caesarean section category was defined as caesarean section performed based on a recorded medical indication. The indicated categories included: repeat caesarean section, non-reassuring foetal heart tracing (NRFHT), failure to progress or cephalopelvic disproportion (CPD) in labour, and previous uterine surgery. NRFHT criteria were consistent with definitions in Williams Obstetrics (23rd Edition).

Other common indications in China which are not necessarily globally accepted indications for caesarean section such as: preeclampsia/eclampsia/HELLP, oligohydramnios, third trimester bleeding (previa/accrete/abruption placenta), multiple gestation, suspected macrosomia, and others (for indications with low frequencies) were included in the “indicated” category in this study. For these diagnoses there was not sufficient clinical information in the database to determine which of these cases would meet internationally accepted criteria. For example the severity of preeclampsia, the etiology of the third trimester bleeding (placenta previa versus abruption placenta), or details of the multiple gestation, higher order pregnancies, twin presentation and concordance were unknown.

The non-indicated category was defined as a primary caesarean section documented by the physician based solely on maternal request in the absence of any maternal or foetal medical indications or physician documented “indications” that show a provider preference but non-standard indication. These included: diagnosis of cephalopelvic disproportion prior to the onset of labour based on clinical pelvimitry and/ or estimated foetal weight, maternal request for their ages greater than or equal to 35 years old, maternal request for precious foetus, precious foetus defined as in vitro pregnancy or poor obstetric history (i.e. prior foetal death, neonatal death, chromosomal or structural abnormality), isolated premature rupture of membranes without foetal heart rate abnormalities, nuchal cord seen on ultrasound without foetal heart rate abnormalities, severe myopia, request for concomitant myomectomy or ovarian cystectomy, or other (isolated chronic hypertension; gestational hypertension; diabetes mellitus without macrosomia, etc.).

Definition of other terms utilized in the study based on Chinese terms: operative vaginal delivery includes forceps delivery, vacuum extraction delivery and breech extraction. Preterm birth was defined as delivery between 24 and 36 6/7 weeks in gestation. Foetal Growth Restriction refers to a foetus with a birth weight less than the 10th percentile. Previous uterine surgery was defined as previous uterine surgery, such as myomectomy, excluding prior caesarean section; malpresentation includes breech presentation, face presentation, transverse lie, and unstable lie. Gestational Diabetes Mellitus refers to abnormal glucose tolerance occurring or initially found during pregnancy by oral glucose tolerance test with any single blood glucose at or above the fasting,1 and 2 h values of 5.1, 10.0, 8.5 mmol/L (92, 180,153 mg/dl), respectively. Obesity in China is defined as body mass index (BMI) ≥ 28 [13]. Suspected macrosomia defined as an estimated foetal weight >4000 g based on ultrasound or Leopold’s maneuvers, regardless of diabetic status. Oligohydramnios represented amniotic fluid index (AFI) ≤5 cm regardless of foetal growth.

### Statistics analysis

Statistics were performed using SPSS statistics software version 18.0. Data was presented as percentages or median with inter-quartile range. Mann–Whitney *U* test was used for the comparison of maternal age and maternal education among groups. Pearson’s chi-square test of independence was used for comparing other frequencies.

## Results

### Modes of delivery

**Fig. 1:**
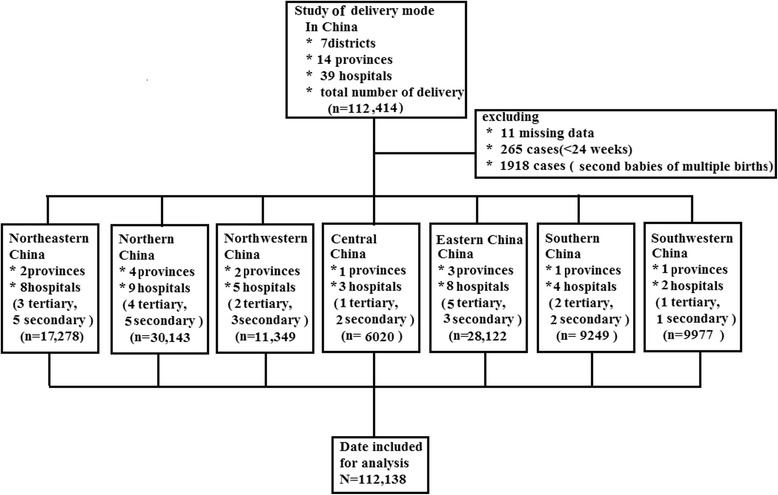
A retrospective analysis that was performed on112414 babies, covering 39 hospitals of different levels in mainland China

A total of 112,414 deliveries occurred during the study period, and 112,138 (99%) deliveries were used for this survey. Two hundred seventy-six participants were excluded because of missing information (11 cases), or giving birth prior to 24-week gestation (265 cases) (Fig. 1). The overall caesarean section rate among the study participants was 54.5%.

The tertiary hospitals comprised 71% (79, 631/112,138) of the study deliveries. In the tertiary hospitals, the caesarean section rate was 55.9% (44, 535/79, 631), significantly higher than that of the secondary hospitals (50.9%, 16, 549/32, 507). Caesarean section with indications accounted 61.6% of caesarean sections. Non-indicated caesarean section accounted for 20.9% of all deliveries or 38.4% of all caesarean sections. Although the secondary level hospitals had lower overall caesarean section rates, there were more non-indicated caesarean sections (48.9% of caesarean sections (8,092/16, 549)) compared to tertiary care hospitals (34.5% (15,382/44535) (Table 1).

**Table 1:** Detailed information about the rates of different deliveries at the 39 hospitals across China

Regions	Cesarean delivery		Overall rates of CS (%)	Vaginal Delivery		Number of deliveries (n)	Number of hospitals (n)
	With indications (%)	Without indications (%)		Spontaneous (%)	Operative (%)^a^		
Tertiary hospitals	29153 (36.6)	15382 (19.3)	44535 (55.9)	33896 (42.6)	1200 (1.5)	79631	20
secondary hospitals	8457 (26.0)	8092 (24.9)	16549 (50.9)	15838 (48.7)	120 (0.4)	32507	19
Total	37610 (33.5)	23474 (20.9)	61084 (54.5)	49734 (44.4)	1320 (1.2)	112138	39

^a^Operative vaginal delivery includes forceps delivery, vacuum extraction delivery and breech extraction

The overall operative vaginal delivery rate was 1.2% with 90.9% (1200/1320) of the operative vaginal deliveries performed in the tertiary level hospitals. The operative vaginal delivery rate in the secondary level hospitals was 0.4% (120/32,507).

### Population characteristics

The characteristics of women by the level of hospitals are presented in Table 2. The average age of the parturients was 28 years old. Women delivering in the tertiary hospitals were significantly older than women who delivering in the secondary hospitals (*P* < 0.001). Women who delivered in the tertiary hospitals had a significantly higher level of education than women who delivered in the secondary hospitals (*P* < 0.001). The overall obesity prevalence was 17.4%. Significantly more women in the tertiary hospitals were obese (19.0%), compared to those in the secondary hospitals (13.2%, *P* < 0.001). Overall, 81.1% of all deliveries were women having their first child. There were more primiparous women in the tertiary hospitals (83.9%, 66,827/79,631) than in the secondary hospitals (74.3%, 24,144/32,507). Male infants accounted for 54.7% of all deliveries. The rate of mothers carrying a male-foetus in the tertiary hospitals was lower than that in the secondary hospitals (54.3% vs. 55.8%,*P* < 0.001). Compared to secondary hospitals, pregnancy complications were more likely in women in the tertiary hospitals, including hypertension disorder complicating pregnancy (HDCP), gestational diabetes, PROM, preterm birth, multiple foetus or late pregnancy bleeding.

**Table 2:** Characteristics of study populations

Variable	All (*n* = 112138) *n* (%)	Tertiary hospital (*n* = 79631) *n* (%)	Secondary hospital (*n* = 32507) *n* (%)	*X* ^2^	*p*
Maternal age (years)					<0.001
(Years)		29 (26–31)	26 (23–30)		
≤ 24	25734 (23.1)	12950 (16.4)	12784 (39.7)		
25–34	74355 (66.8)	57536 (72.8)	16819 (52.2)		
≥ 35	11197 (10.1)	8583 (10.9)	2614 (8.1)		
Maternal education level					<0.001
College or above	55473 (51.3)	46093 (59.0)	9380 (31.3)		
High school	29935 (27.7)	20411 (26.1)	9524 (31.8)		
Primary school	22468 (20.8)	11442 (14.6)	11026 (36.8)		
Illiteracy	258 (0.2)	214 (0.3)	44 (0.1)		
Residents	77661 (69.3)	56595 (71.1)	21066 (64.8)	425.80	<0.001
Obesity	15980 (17.4)	12512 (19.0)	3468 (13.2)	1584.92	<0.001
Primiparous	90971 (81.1)	66827 (83.9)	24144 (74.3)	1403.10	<0.001
Previous bad obstetric history	1084 (1.0)	952 (1.2)	132 (0.4)	150.28	<0.001
Male infants	61345 (54.7)	43028 (54.3)	18137 (55.8)	28.84	<0.001
Multiple fetus	1918 (1.7)	1771 (2.2)	147 (0.5)	431.05	<0.001
GDM/DM	5344 (4.8)	5024 (6.3)	320 (1.0)	1442.08	<0.001
HDCP	5357 (4.8)	4594 (5.8)	763 (2.3)	594.21	<0.001
Preeclampsia/eclampsia	4137 (3.7)	3650 (4.6)	487 (1.5)	618.52	<0.001
PROM	17078 (15.2)	13965 (17.5)	3113 (9.6)	1133.14	<0.001
Preterm birth	9014 (8.0)	8156 (10.2)	858 (2.6)	1805.02	<0.001
Late pregnancy bleeding	2364 (2.1)	2127 (2.7)	237 (0.7)	421.85	<0.001
oligohydrammios	4200 (3.7)	2938 (3.7)	1262 (1.6)	2.37	0.12
Fetus weight ≥ 4000 g	7366 (6.7)	5075 (6.5)	2291 (7.1)	14.38	<0.001

Quantitative variables of age are expressed as median (25th percentile–75th percentile)

852 missing cases for maternal age; 4004 missing cases for maternal education, 20187 missing cases for maternal BMI;, 2071 missing cases for neonatal weight

*IVF-ET* In Vitro Fertilization Pre-Embryo Transfer, *PROM* Preterm Rupture Of Membranes, *HDCP* Hypertension Disorder Complicating Pregnancy

### Indications for caesarean section

Among 61,084 caesarean sections, 7.8% (4,783) were repeat caesarean section and 92.2% (56,301) were primary caesarean section. Analysis of the primary caesarean section showed that 53.7% were with indications and 38.4% were without indications (Table 3). Overall caesarean section on maternal request was the most common reason (22% of all caesarean sections).

**Table 3:** The indications for cesarean by regions

Indications	Total (% of 61084)	Total (% of 112138)	Tertiary hospital (% of 79631)	Secondary hospital (% of 32507)	*X* ^2^ value	*P* value
Total Cesarean Delivery	100	61084 (54.5)	44535 (55.9)	16549 (50.9)	234.35	<0.001
Repeat Cesarean Delivery	7.8	4783 (4.3)	3640 (4.6)	1143 (3.5)	62.91	<0.001
Primary Cesarean Delivery	92.2	56301 (50.2)	40895 (51.4)	15406 (47.4)	145.00	<0.001
Non-indicated	38.4	23474 (20.9)	15382 (19.3)	8092 (24.9)	433.71	<0.001
Cesarean Delivery on maternal demand	21.9	13367 (11.9)	7957 (10.0)	5410 (16.6)	972.35	<0.001
Other non-indicated Cesarean Delivery	16.5	10107 (9.0)	7425 (9.3)	2682 (8.3)	32.45	<0.001
Indicated Cesarean Delivery	53.7	32827 (29.3)	25513 (32.0)	7314 (22.5)	1014.56	<0.001
NRFHT	12.8	7840 (7.0)	5736 (7.2)	2104 (6.5)	19.00	<0.001
FTP/CPD (in labor)	8.8	5373 (4.8)	4006 (5.0)	1367 (4.2)	34.48	<0.001
Malpresentation	5.7	3500 (3.1)	2811 (3.5)	689 (2.1)	151.88	<0.001
Suspected macrosomia	5.6	3427 (3.1)	2463 (3.1)	964 (3.0)	1.27	0.26
Preeclampsia/ eclampsia	4.3	2650 (2.4)	2356 (3.0)	294 (0.9)	422.18	<0.001
Oligohydrammios	3.9	2393 (2.1)	1807 (2.3)	586 (1.8)	24.06	<0.001
Late pregnancy bleeding	2.7	1637 (1.5)	1476 (1.9)	161 (0.5)	296.05	<0.001
Prior uterine surgery	2.5	1554 (1.4)	1244 (1.6)	310 (1.0)	62.56	<0.001
Multiple fetus	2.1	1280 (1.1)	1178 (1.5)	102 (0.3)	277.90	<0.001
FGR	2.0	1194 (1.1)	629 (0.8)	565 (1.7)	197.01	<0.001
Other medical disease	1.2	727 (0.6)	694 (0.9)	33 (0.1)	211.49	<0.001
Other indications	2.0	1252 (1.1)	1113 (1.4)	139 (0.4)	196.77	<0.001

In the overall study population, of the top 11 indications for caesarean section, NRFHT was the most common medical indication (12.8% of all caesarean sections), followed by cephalopelvic disproportion/ failure to progress (8.8% of all caesarean sections), and malpresentation (5.7% of all caesarean sections). Other indications included suspected macrosomia (5.6%), preeclampsia/eclampsia (4.3%), oligohydramnios (3.9%), late pregnancy bleeding (2.7%), prior uterine surgery (2.5%), multiple foetuses (2.1%) and FGR (2.0%) (Table 3).

There were differences in indications for caesarean section between the different level of hospitals. In the tertiary hospitals, of the 79, 631deliveries, there was a higher percent of caesarean section for NRFHT or cephalopelvic disproportion/ failure to progress (*P* < 0.001). The caesarean section rate for pregnancy complications (such as HDCP, malpresentation, oligohydramnios, late pregnancy bleeding, prior uterine surgery and multiple foetuses) in the tertiary hospitals was higher than in the secondary hospitals (*P* < 0.001).

Of the top 7 non-indications for caesarean section, the top three reasons recorded were: maternal request without any reason (56.9% of caesarean sections,13,367/23,474),CPD prior to labour (20.2% of caesarean sections,4756/23,474) and maternal request for age ≥35 years (11.2% of caesarean sections, 2630/23,474) which accounted for 88.4% of the non-indicated caesarean sections, followed by maternal request for precious baby (3.2%), request for myomectomy (2.3%), nuchal cord (1.5%), isolated PROM (1.4%), and severe myopia (1.0%) (Table 4).

**Table 4:** The non-indications for cesarean by regions

Indications	Total (% of 23474)	Total (*n* = 112138)	Tertiary hospitals (% of 79631)	Secondary hospital (% of 32507)	*X* ^2^ value	*P* value
Maternal request without any reason	56.9	13367 (11.9)	7957	5410	972.35	<0.001
CPD prior to labor	20.2	4756 (4.2)	2904	1852	238.96	<0.001
Maternal request for Age >35 (multip + primip)	11.2	2630 (2.3)	2097	533	99.53	<0.001
Materal request for precious baby	3.2	761 (0.7)	662	99	95.04	<0.001
Maternl request for myomectomy	2.3	540 (0.5)	503	37	129.17	<0.001
Nuchal Cord	1.5	342 (0.3)	306	36	55.80	<0.001
Isolated: PROM	1.4	336 (0.3)	292	44	41.35	<0.001
Severe myopia	1.0	225 (0.2)	221	4	81.09	<0.001
TOTAL	100	23474 (20.9)	15382	8092	433.71	<0.001

The caesarean section rate for maternal request without any reason was much higher in the secondary hospitals than in the tertiary hospitals (*P* < 0.001), as well as the caesarean section rate for CPD prior to labour (*P* < 0.001). The caesarean section rate for other non-indications, such as maternal request for older age or precious baby was higher in the tertiary hospitals than that in the secondary hospitals (*P* < 0.001) (Table 4).

## Discussion

This study of 112,138 births is one of the largest-scale surveys including indications for delivery conducted in China to date, covering three geographical regions, and focusing on non-indicated caesarean sections. It showed an overall caesarean section rate of 54.5% in 2011 which is substantially higher than the 46.2% found in the 2008 WHO study [1]. As an example, at the Beijing Obstetrics and Gynecology Hospital, the largest tertiary specialty hospital in Beijing, the caesarean section rate steadily increased from 19.5% in 1980–1984, to 25.4% in 1985–1988, then 35.3% in 1989–1992 [14], and the rate was the highest ever recorded at 47.9% in 2011 in this survey. The high caesarean section rate in China is concerning and efforts to analyze caesarean section rates and indications are important first steps in designing programs to decrease these rates.

In this study, 38.4% of all caesarean section were non-indicated, with 85.6% scheduled and performed before labour. Non-indicated caesarean section in this study accounted for 20.9% of deliveries compared to only 11.2% in the 2008 WHO study.

In China as early as 1996, caesarean section on maternal request or caesarean section for social factors received attention from researchers [15]. Previous studies also demonstrated that caesarean section on maternal request or caesarean section for “social factors” accounted for 35.9–46.2% of all caesarean section in certain areas of China [7]. In this study 11.9% of deliveries were caesarean section on maternal request compared to rates estimated at 2.5% in the USA or 1-2% in the United Kingdom. Caesarean section on maternal request was the most common recorded reason for caesarean section in this study. The non-indicated caesarean section rate varied greatly by level of facility and region and was much higher in the secondary level hospitals than in the tertiary level hospitals. This suggested that clinical practice patterns affect the number of caesarean sections performed. In China in 2011, there was not a gatekeeper system or a pre-operative view required for caesarean section [16] so if a woman requested a caesarean section, some hospitals were more likely to comply.

Prior studies in China have reported that fear of pain and perceived better health for the child and mother are the main reasons women request caesarean section [17–19]. In China in 2011, most public hospitals were unable to routinely offer pain relief or epidurals in labour making the fear of pain a real concern. Emotional and nursing support for women in labour requires adequate nurse to patient staffing ratios, which may be lacking with the current system.

CPD diagnosed prior to labour was the second most common coded reason for non-indicted caesarean section, accounting for 20% of the non-indicated caesarean section or 4.2% of total deliveries. Even in the tertiary hospitals, CPD prior to labour accounted for 18.9% of the non-indicated caesarean section or 3.6% of total deliveries. In most hospitals in China routine clinical pelvimitry is performed antenatally. Previous Chinese studies classifed this as a medical indication for caesarean section. Despite abandonment of this practice in the USA and UK due to its poor predictive value for failure to progress in labour, in China it is a common physician documented reason for caesarean section.

In China, tertiary level hospitals often provide leadership in clinical teaching, research, and development of clinical guidelines. Physicians from tertiary hospitals may be considered more informed than those at secondary level hospitals, but this study suggests a wide gap between the tertiary level physician practice and international guidelines. The development of standardized evidence-based guidelines in obstetrics may help obstetricians decrease the number of non-indicated caesarean section.

In China, high risk pregnant women delivered more often in the tertiary hospitals. The higher overall caesarean section rate in tertiary level hospitals compared to secondary level hospitals may be partially explained by the higher risk patient mix at the tertiary level hospitals.

In this study 33.5% of deliveries were caesarean section with documented indications, with 29.3% of primary caesarean section with indications. Among the indicated caesarean sections, the most common diagnosis was NRFHRT or foetal distress (12.8% of all caesarean section, 7.0% of all deliveries). This is consistent with a previous study of teaching hospitals in China [20] and is higher than that in the United States (4.0-5.9%) [21, 22]. The rate for this indication was much higher in the tertiary hospitals than in the secondary hospitals in this study. NRFHT can be a relatively subjective indication for caesarean section [22, 23]. In this study NRFHT was diagnosed before labour 36.0% (2824/7840) of the time. This finding may be partially explained by the common overuse of technology in low risk pregnancies in China. For example, in many hospitals, the standard of care is to perform weekly non-stress tests (NSTs) in all healthy pregnant women in the third trimester. The positive predictive value of these tests is poor when used in low risk populations. Isolated abnormal umbilical cord dopplers or nonspecific electronic foetal monitor findings may result in unnecessary caesarean section for “foetal distress”.

The non-indicated caesarean section rate would be even higher if some diagnoses that we included in the “indicated” category such as preeclampsia/eclampsia/HELLP, oligohydramnios, third trimester bleeding (previa/accrete/abruption placenta), multiple gestation, and suspected macrosomia were further analyzed and re-categorized since they are not globally accepted and evidence based indications for caesarean section. In this analysis we were unable, for example, to discern which pregnancies had placenta previa (an accepted caesarean section indication) versus an abruptio placenta without intrauterine foetal death (not a caesarean section indication) or which multiple gestation cases had a non-vertex presenting twin, higher order multiples or non-concordant breech second twins (all accepted caesarean section indications). Thus with rigorous auditing of the “indicated” category of cases, many would be reclassified as “non-indicated”, raising the overall non-indicated caesarean section rate.

Isolated oligohydramnios is also not an evidence based indication for caesarean section.

Consistent with previous research in China, our study showed that isolated oligohydramnios was one of the major indications for caesarean section in China, accounting for 3.9% of all caesarean section. A prior study involving 2326 women of rural eastern China showed that, during antenatal care, 46.8% women received at least 3 ultrasound scans and the maximum number reached 11 [8]. It showed a statistically significant association between the number of antenatal ultrasound scans and caesarean section rates. A growing body of evidence demonstrates that isolated oligohydramnios in the absence of other maternal or foetal risk factors is not associated with adverse perinatal outcomes [24]. Caesarean section for preeclampsia could also be reassigned to non-indicated caesarean section if we were able to analyze the cases with more clinical information.

In this study, the overall operative vaginal delivery rate was very low at 1.2% and even lower in the secondary hospitals. This is consistent with Wang’s report, which surveyed 887 health facilities in the year 2002 and reported a national caesarean section rate of 38.0% and an extremely low operative vaginal delivery rate (5.5%) [25]. Low operative vaginal delivery rates may be due to pressure on the caregivers to practice “defensive medicine” and lack of training and experience with the techniques. Caesarean section for malpresentation accounted for 3.2% of all deliveries. Perhaps due to the same pressures few hospitals offer external cephalic versions.

Numerous studies of caesarean section have shown a strong association between increased maternal education, older maternal age and low parity and an increased risk of caesarean section [6, 15, 16]. In China, tertiary hospitals are located in big cities, treating mainly urban patients or rural patients referred from secondary and primary hospitals. Secondary and primary hospitals are usually in moderate or small cities, treating patients from towns or rural areas. In this study, the tertiary level hospital parturients were older, more educated, more likely to be having their first child later, pregnancy complications and indicated caesarean section.

The major strength of this study is that it is one of the largest-scale surveys conducted in China to date, covering three geographical regions, and focusing on non-indicated caesarean section in China. The biggest limitation of this study is that the facilities were not randomly selected, which may have introduced selection bias. Thus the caesarean section rates should not be regarded as representative for the entire country, nor for all the regions. Furthermore, our survey included only the secondary and tertiary hospitals, so our results cannot be generalized to smaller facilities. Second, prior studies show an association between insurance coverage and caesarean section but this cannot be analyzed since insurance data was not recorded in our survey. Third, we have no data on labour management including: induction of labour, augmentation of labour, or the availability of anesthesia. Furthermore, In the paper based on the same database, which we published in Chinese in 2014 [26], we put the indictions, such as cephalopelvic disproportion prior to labor, Age >35,etc. into indicated caesarean section as we used to do, it showed that the non-indicated caesarean section rate was 24.553%, was lower than that of this article, we have corrected in this article. Even so, there is some misclassification bias given that many of the caesarean section classified as “indicated” would likely be “non-indicated” if we had more clinical data on the cases. This suggests that we have underestimated the number of non-indicated caesarean sections.

The ideal caesarean section rate is not known. One ecological study using longitudinal data in 159 countries showed caesarean section rates higher than around 10% at the population level are not associated with decreases in maternal and neonatal mortality rate [27]. Another suggested that rates up to approximately 19% were associated with lower maternal and neonatal mortality rates in WHO member states [28]. Regardless, the caesarean section rate in China in this study of 54.5% is well above accepted target rates with at least 38% that were non-indicated. The motivations for providers are to perform caesarean sections for non-medical, vague or subjective indications is a complex issue. A broader perception of caesarean section as safe has been raised as a possibility. The use of globally recognized indications that are evidence- based to create clinical obstetric guidelines could standardize diagnosis and treatment in pregnancy and result in fewer unnecessary caesarean section at tertiary and secondary level hospitals. Additionally, medical malpractice reform and focus on the birth experience may be a way to reduce caesarean section rates. Analysis of maternal and perinatal outcomes associated with mode of delivery in China needs further study. With the end of the One Child policy, as of January 2016, the risks associated with non-indicated caesarean section will likely increase as more women are allowed and choose to have a second child. This makes it imperative to reduce the number of unnecessary primary caesarean sections.

## Conclusions

Caesarean section on maternal request accounts for a large portion of China’s high caesarean section rate, especially in the secondary hospitals. More guidelines and bulletins are urgently needed to standardize the diagnosis and treatment of diseases nationally among different level of hospitals. On the other side, legislation to protecting doctors to do the appropriate treatment rather than defense treatment is still the important things to do.

## Abbreviations

AFI: Amniotic fluid index
BMI: Body mass index
CI: Confidence intervals
CPD: Cephalopelvic disproportion
DM: Preexisting diabetes mellitus
FGR: Foetal growth restriction
GDM: Gestational diabetes mellitus
HDCP: Hypertension disorder complicating pregnancy
IVF-ET: In vitro fertilization pre-embryo transfer
NRFHT: Non-reassuring foetal heart tracing
OR: Odds ratios
PROM: Preterm Rupture Of Membranes
WHO: World Health Organization
